# Oxford NOTECHS II: A Modified Theatre Team Non-Technical Skills Scoring System

**DOI:** 10.1371/journal.pone.0090320

**Published:** 2014-03-04

**Authors:** Eleanor R. Robertson, Mohammed Hadi, Lauren J. Morgan, Sharon P. Pickering, Gary Collins, Steve New, Damien Griffin, Peter McCulloch, Ken C. Catchpole

**Affiliations:** 1 Quality, Reliability, Safety and Teamwork Unit, Nuffield Department of Surgical Sciences, University of Oxford, Oxford, United Kingdom; 2 Warwick Orthopaedics, Warwick Medical School, University of Warwick, Coventry, United Kingdom; 3 Centre for Statistics in Medicine, University of Oxford, Oxford, United Kingdom; 4 Saïd Business School, University of Oxford, Oxford, United Kingdom; 5 Department of Surgery, Cedars-Sinai Medical Center, Los Angeles, California, United States of America; Institutes for Behavior Resources and Johns Hopkins University School of Medicine, United States of America

## Abstract

**Background:**

We previously developed and validated the Oxford NOTECHS rating system for evaluating the non-technical skills of an entire operating theatre team. Experience with the scale identified the need for greater discrimination between levels of performance within the normal range. We report here the development of a modified scale (Oxford NOTECHS II) to facilitate this. The new measure uses an eight-point instead of a four point scale to measure each dimension of non-technical skills, and begins with a default rating of 6 for each element. We evaluated this new scale in 297 operations at five NHS sites in four surgical specialities. Measures of theatre process reliability (glitch count) and compliance with the WHO surgical safety checklist were scored contemporaneously, and relationships with NOTECHS II scores explored.

**Results:**

Mean team Oxford NOTECHS II scores was 73.39 (range 37–92). The means for surgical, anaesthetic and nursing sub-teams were 24.61 (IQR 23, 27); 24.22 (IQR 23, 26) and 24.55 (IQR 23, 26). Oxford NOTECHS II showed good inter-rater reliability between human factors and clinical observers in each of the four domains. Teams with high WHO compliance had higher mean Oxford NOTECHS II scores (74.5) than those with low compliance (71.1) (p = 0.010). We observed only a weak correlation between Oxford NOTECHS II scores and glitch count; r = −0.26 (95% CI −0.36 to −0.15). Oxford NOTECHS II scores did not vary significantly between 5 different hospital sites, but a significant difference was seen between specialities (p = 0.001).

**Conclusions:**

Oxford NOTECHS II provides good discrimination between teams while retaining reliability and correlation with other measures of teamwork performance, and is not confounded by technical performance. It is therefore suitable for combined use with a technical performance scale to provide a global description of operating theatre team performance.

## Introduction

The evaluation of team non-technical skills has become important in research on surgical safety because of the evidence that teamwork glitches, communication failures, cultural and hierarchal barriers contribute to safety failures[1]–[4]. Comparisons between the work environments of airline cockpits and operating theatres encouraged researchers to apply techniques originally developed to evaluate team interaction within the aviation industry to situations in healthcare[5]–[7].

Non-technical skills assessment tools adapted from aviation have been used in the operating theatre to understand the influence of behaviour on outcome. A number of methods have been developed for assessing teamwork skills in operating theatres, based on direct observation or video analysis[1], [8]–[14]. The development of the first NOTECHS assessment method was conducted via a European collaboration[15] which has since been followed by further developments. OTAS[16]; Oxford NOTECHS[14], OSTAS[17] and EPOC[18] provide whole team assessments; ANTS[10], NOTSS[19] and SPLINTS[20] focus on sub-team performance. The methodology underpinning the Oxford NOTECHS tool maps directly to the original methods used in the aviation industry via a carefully planned adaptation for use with surgical teams[14], [21]. It has since been successfully applied in a range of surgical settings examining the effects of Crew Resource Management (CRM)-based training courses on surgical teams[22], [23].

As the complexity of surgical team working becomes clearer, so the demands for more sophisticated methods of measurement have followed. The validity and reliability of the Oxford NOTECHS system was demonstrated in live theatre environments[14], but a number of imperfections were noted in discussion and through experience. The system tended to group results close to the median, and therefore had a suboptimal capacity to discriminate between teams whose performance was not extreme.

As part of a wider project aimed at extensively examining the effects of a theatre based teamwork training intervention, we took the opportunity to restructure the original Oxford NOTECHS tool (Oxford NOTECHS II). The purpose of this re-design was to develop a tool with improved scalability, enabling a richer understanding on the impact of quality improvement interventions. This study describes and evaluates the new scale (Oxford NOTECHS II).

## Methods

### Ethics Statement

Ethics Committee approval was obtained for this study (NRES Committee South Central - Oxford A REC 09/H0604/39). Hospital management and all theatre staff were fully informed of the study and provided written consent to take part during the observation period.

Oxford NOTECHS II is built on extensive work in developing, evaluating, and validating the original Oxford NOTECHS[14]. The final version was arrived at through group discussion between members of the original (PM, KC) and new group members (ER, LM, MH and SP), statistical modelling (GC) followed by iterative small-scale testing. The four original Oxford NOTECHS domains of: leadership and management (L&M); teamwork and cooperation (T&C); problem-solving and decision-making (PS & DM); and situation awareness (SA) remain unchanged. The behavioural markers for each of the Oxford NOTECHS II parameters are largely unchanged from before (Table 1). There is no alteration to the consideration of the theatre team as three sub-teams: surgical (operating and assisting surgeons); anaesthetic (anaesthetists and anaesthetic nurses/practitioners) and nursing (scrub and non-anaesthetic circulating nurses and practitioners).

**Table 1:** Operating theatre team Non-Technical Skills (NOTECHS) assessment tool[14].

**Leadership and management**	
Leadership	Involves/reflects on suggestions/visible/accessible/inspires/motivates/coaches
Maintenance of standards	Subscribes to standards/monitors compliance to standards/intervenes if deviation/deviates with team approval/demonstrates desire to achieve high standards
Planning and preparation	Team participation in planning/plan is shared/understanding confirmed/projects/changes in consultation
Workload management	Distributes tasks/monitors/reviews/tasks are prioritised/allots adequate time/responds to stress
Authority and assertiveness	Advocates position/values team input/takes control/persistent/appropriate assertiveness
**Teamwork and co-operation**	
Team building/maintaining	Relaxed/supportive/open/inclusive/polite/friendly/use of humour/does not compete
Support of others	Helps others/offers assistance/gives feedback
Understanding team needs	Listens to others/recognises ability of team/condition of others considered/gives personal feedback
Conflict solving	keeps calm in conflicts/suggests conflict solutions/concentrates on what is right
**Problem-solving and decision-making**	
Definition and diagnosis	Uses all resources/analytical decision making/reviews factors with team
Option generation	Suggests alternative options/asks for options/reviews outcomes/confirms options
Risk assessment	Estimates risks/considers risk in terms of team capabilities/estimates patient outcome
Outcome review	Reviews outcomes/reviews new options/objective, constructive and timely reviews/makes time for review/seeks feedback from others/conducts post treatment review
**Situation awareness**	
Notice	Considers all team elements/asks for or shares information/aware of available of resources/encourages vigilance/checks and reports changes in team/requests reports/updates
Understand	Knows capabilities/cross checks above/shares mental models/speaks up when unsure/updates other team members/discusses team constraints
Think ahead	Identifies future problems/discusses contingencies/anticipates requirements

The differences from the original Oxford NOTECHS are the use of an eight point instead of a four-point scale for dimensions; assigning all teams a baseline score of 6, a behavioural marker of ‘consistently maintaining an effective level of patient safety and teamwork’(Table 2), from which subsequent observations could result in deviation upwards or downwards in response to observable behavioural markers[14].

**Table 2:** Behavioural parameters of Oxford NOTECHS II.

Behaviour	Frequency	Oxford NOTECHS II score
Compromises patient safety and effective team work	Consistently	1
	Inconsistently	2
Could directly compromise patient safety and effective team work	Consistently	3
	Inconsistently	4
Maintains an effective level of patient safety and teamwork	Inconsistently	5
	Consistently	6
Enhances patient safety and effective teamwork	Inconsistently	7
	Consistently	8

### Training of observers

Observations of intra-operative process and non-technical skills were performed real-time in the operating theatre by two observers, one being a clinical and one a human factors (HF) specialist. The clinical observers were either surgical trainees (ER & MH) or operating department practitioners (JM) with >1 years theatre experience. The HF specialists had at least an undergraduate qualification in human factors (LM, SP and LB). The clinical observers gained experience of HF principles from in-house lectures and literature reviews and the HF observers gained experience of the theatre environment from theatre observational practice and mentoring by clinical observers. All observers trained in the use of the Oxford NOTECHS II over a one month period with self-study and group practice sessions using video recordings of operating teams in simulated settings.

### Logistics of scoring Oxford NOTECHS II

The three theatre sub-teams are each scored on four parameters (L&M, T&C, PS & DM and SA) resulting in a total of twelve scores. The final scores were calculated at the end of the operation (during suturing/applying dressings) and entered into procedure-specific data collection books in an independent manner[24]. Once the operation was complete, each observer's scores were individually entered on a secure database.

The data for this study was collected as part of a multi-centre observational study at five hospitals from three NHS trusts, the Safer Delivery of Surgical Services Programme (S3). The hospitals comprised: one district general hospital, three teaching hospitals and one tertiary referral centre. A variety of surgical specialities were selected for observation including: elective orthopaedic; trauma orthopaedics; plastic surgery and vascular/general surgery. Within each surgical speciality a range of surgical interventions were selected for inclusion in the study based on frequency, and operative lists were targeted with the highest proportion of this pre-determined case mix (Table 3). This was therefore a large convenience sample with predefined characteristics.

**Table 3:** Surgical case mix.

Surgical Speciality	Types of operation
Elective Orthopaedics	Primary and revision knee and hip arthroplasty and arthroscopic procedures
Trauma Orthopaedics	Manipulation of fractures and dislocations under anaesthetic, open reduction and either internal fixations or hemi-arthroplasty procedures
Vascular Surgery	Arterial bypass, endarterectomies and hernia repair
Plastic Surgery	Excision of benign and malignant lesions with a range of closure techniques including free flaps and upper limb and nerve surgery

### Validation

Alongside non-technical skills rating, observers collected information on the technical performance of the team and the quality and completeness of the WHO time-out procedure. When the WHO time-out was attempted, observers critiqued process quality using three binary (yes/no) parameters: was all information communicated; were all team members present and was there active participation. Observers recorded these parameters independently, then discussed their findings at the conclusion of the case to reach agreement on a joint final score[25]. Intra-operative process disruptions (e.g. distractions, interruptions, planning and preparation issues) were evaluated using the glitch method[26]. The associations between both the glitch count and WHO time-out performance and Oxford NOTECHS II scores were subjected to exploratory statistical analysis to provide independent validation of the new scale, in addition to that provided for the original Oxford NOTECHS[14].

To further explore the relationship between Oxford NOTECHS II scores and glitch count, a Delphi experiment was undertaken within the research group. The researchers were asked to predict the relationship between Oxford NOTECHS II scores and the 14 glitch sub-categories[26]. This experiment was undertaken in two rounds, with researchers ranking the likelihood of correlation of each sub-category with Oxford NOTECHS II. At the end of the second round, four sub-categories were grouped as ‘likely to correlate’, six as ‘may or may not correlate’ and four as ‘will not correlate’ (Table 4). A separate analysis of the association between each of these sub-categories and Oxford NOTECHS II was carried out.

**Table 4:** Results of the team Delphi experiment, categorising the predicted relationship between Oxford NOTECHS II and glitch category.

Delphi results	Glitch categories with Delphi ranking
Likely to correlate	1. Planning and preparation
	2. Communication
	3. Absence
	4. Process deviation
Potentially correlate	5. Training
	6. Distractions
	7. Health and safety
	8. Workspace
	9. Maintenance
	10. Maintenance equipment
Will not correlate	Slips
	Environment
	Equipment design
	Patient related factors

### Statistical methods

Agreement between observers (clinical and HF) for each dimension of the Oxford NOTECHS II instrument was assessed using agreement and the intraclass correlation coefficient. Mean Oxford NOTECHS II scores were compared for operations with high and low WHO time-out scores[25] using the t-test. Differences between mean Oxford NOTECHS II scores for different specialities and sites were examined using analysis of variance. Where differences between specialties or between sites were observed, the t-test was used to explore where the difference lay; no adjustment was made for multiple testing. The correlation between Oxford NOTECHS II scores and glitch rate was explored graphically and quantified by Pearson's correlation coefficient. We considered P values <0.05 to be statistically significant. All statistical analyses were carried out in R.3.0.1.

## Results

### A total of 297 operations were observed, lasting an average of 1 hour 53 minutes. Site A was the primary study site, and so had the largest number of operations (Table 5)

**Figure 1:**
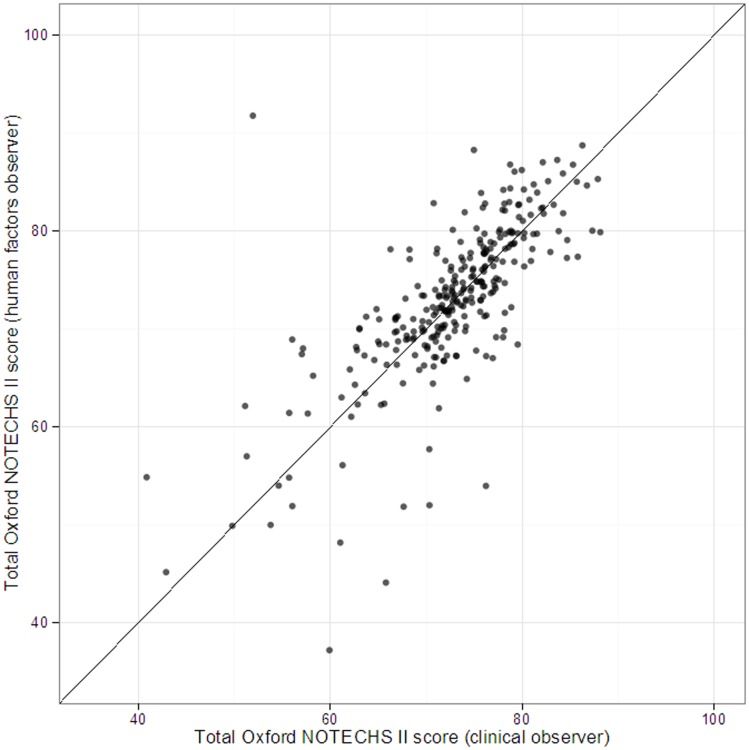
Inter-rater agreement between HF and clinical observers.

An initial live test of inter-rater reliability in 20 elective orthopaedic operations across multiple sites showed good inter-rater agreement[27]. Subsequent analysis on the whole dataset confirmed the maintenance of good inter-rater agreement for total Oxford NOTECHS II score between the HF and clinical observers (Figure 1 and Table 6).

**Table 5:** Number of operations performed by site and speciality.

Number of operations	Site A	Site B	Site C	Site D	Site E	Total specialty
Elective Orthopaedics	n = 101	n = 26	n = 54	n = 30	-	n = 211
Trauma Orthopaedics	-	-	n = 16	-	-	n = 16
Vascular surgery	-	n = 21	-	-	n = 27	n = 48
Plastic surgery	n = 22	-	-	-	-	n = 22
Total per site	n = 123	n = 47	n = 70	n = 30	n = 27	n = 297

**Table 6:** Inter-rater agreement (% agreement, intraclass correlation coefficient).

	Surgical team	Nursing team	Anaesthetic team
Leadership and management	59%, 0.881	59%, 0.777	64%, 0.739
Teamwork and cooperation	55%, 0.757	45%, 0.343	64%, 0.676
Problems solving and decision making	55%, 0.725	63%, 0.397	78%, 0.385
Situational awareness	53%, 0.770	48%, 0.683	56%, 0.675

On sub-category analysis we generally found good levels of agreement as evaluated by weighted intraclass correlation coefficient, but some sub-categories had low values despite the observed agreement being high. This was particularly seen in the Problem Solving and Decision Making category for nursing and anaesthetic teams and the Teamwork and Cooperation category for the nursing team. These findings are largely explained by the high concentration of ratings by the two observers (HF and clinical) around a single Oxford NOTECHS II score.

**Figure 2:**
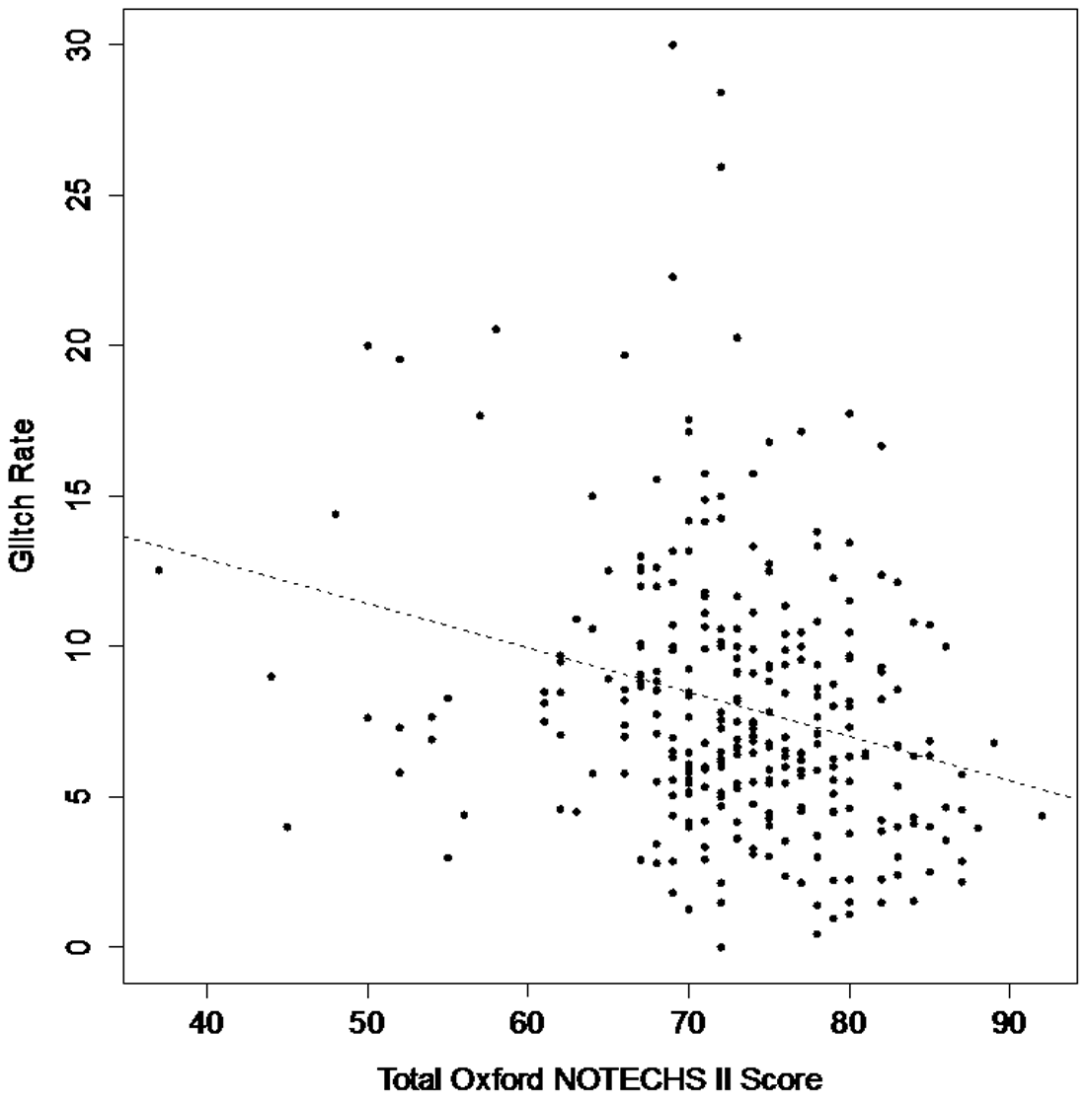
Relationship between glitch rate (total glitch rate per operation per hour) and total Oxford NOTECHS II score.

Oxford NOTECHS II scores were significantly higher (p = 0.01) for teams who met all 3 quality criteria for the WHO time-out (mean Oxford NOTECHS II score 74.5 +/− SD  = 7.3, n = 98) compared to those who met none of them (mean Oxford NOTECHS II score 71.1 +/− SD = 8, n = 54). There was only a weak correlation between NOTECHS II score and glitch rate (r = −0.26, 95% CI −0.36 to −0.15, Figure 2). We found no associations between whole or sub-team Oxford NOTECHS II scores and sub-category glitch rates (Figure 2).

**Figure 3:**
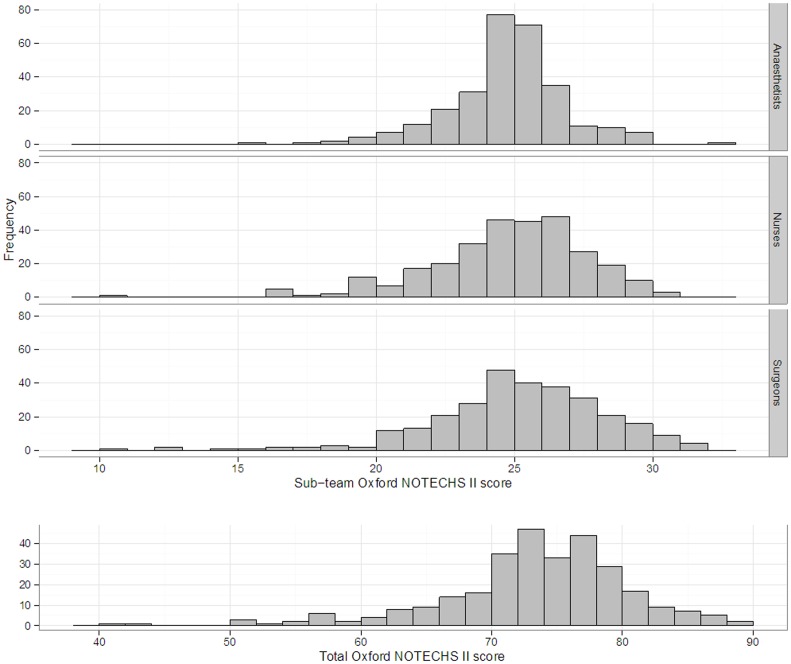
Distribution of NOTECHS II scores for the whole team and the three sub-teams (Anaesthetists, Nurses, Surgeons).

The range and the distribution of Oxford NOTECHS II scores for, the sub-teams and the whole theatre team are displayed in Figure 3. There was no statistically significant difference in mean Oxford NOTECHS II scores between the three theatre sub-teams (surgical team 24.61 (IQR 23, 27); anaesthetic team 24.22 (IQR 23, 26) and nursing team 24.55 (IQR 23, 26)) p = 0.235. The surgical team appeared to vary most and the anaesthetic team the least across all four parameters.

In elective orthopaedic surgery the difference in mean Oxford NOTECHS II at four hospital sites (from 72.85 to 75.23) was not significant (p = 0.643). In vascular surgery, the mean Oxford NOTECHS II score at two hospital sites (from 69.75 to 73.24) was not significant (p = 0.089). Across clinical specialties there was statistically significant heterogeneity in Oxford NOTECHS II score, which ranged from 67.14 in plastic surgery to 73.86 in elective orthopaedic surgery (p = 0.001). This was explained by significant differences between elective orthopaedic and plastic surgery (means 73.86) vs 67.14, difference 6.72, 95% CI 1.48 to 11.97), and between trauma orthopaedics and plastics (means 73 vs 67.14, difference 5.86, 95% CI 0.01 to 11.63).

## Discussion

We set out to develop an improved non-technical skills assessment scale which would provide greater discrimination between the behaviour of theatre teams, as we felt that the tendency of the previous Oxford NOTECHS scale to score most teams within a fairly narrow middle range may have limited its value in studies of the effects of interventions. This was the rationale for doubling the scale and setting a default value close to the equivalent of the median result found in previous studies. Anchoring teams on a starting score of 6 was intended to maintain consistency and frame the observations in the light of positive assumptions about motivation and professionalism [28].

The new scale shows good inter-rater reliability between observers from different backgrounds across five hospital sites and four surgical specialities. This suggests that it may be possible for one observer from either a clinical or HF background to successfully rate non-technical skills using Oxford NOTECHS II after limited training, which would have important resource implications. However further work will be required to demonstrate this.

The final version of the score had improved face validity compared to the original Oxford NOTECHS amongst the research team. We did not alter the fundamentals of the scale, and therefore believe that the validation work performed for Oxford NOTECHS[14] remains relevant. However we studied association between Oxford NOTECHS II and both WHO time-out performance and glitch count, to assess concurrent and construct validity for the new scale. We found a statistically significant association between high quality WHO time-out and higher Oxford NOTECHS II scores. This finding adds support to the hypothesised mechanistic effect of a well performed WHO surgical safety checklist on intra-operative non-technical skills performance. This may be of particular import when teams frequently change – a characteristic of aviation and medical teams.

In contrast to previous work [14], we only found a weak correlation between Oxford NOTECHS II and glitch rate. We studied this association in more detail by developing a consensus grading, from strong to none, for the associations with the new scale we expected to see for different types of glitch, based on our subjective impression of their relationship with the quality of teamwork. Subsequent analysis however showed no association for any of the sub-categories. Technically this may have been because the glitch score represents a substantial re-categorisation of intra-operative events compared to the NOPE (non-operative procedural errors) system used in previous work[29]. Conceptually, it is superficially surprising that this is the case: one could imagine a poorly performing surgical team doing ‘badly’ by having a poor non-technical skills and this being connected to a large number of glitches. However, many glitches are caused by a range of factors exogenous to the team (e.g. interruptions or poor equipment maintenance), whilst the occurrence of glitches may in some cases give teams the opportunity to exercise non-technical skills and achieve Oxford NOTECHS II scores of 7 or 8. This resonates with findings from previous research in the field recognising that resilience and the ability for teams to recover is an important factor in achieving safe care delivery[30], [31]. The relationship between technical and non-technical performance thus appears to be more complex than we had previously anticipated.

We consider that the glitch method measures the effect of the wider system upon the theatre process and the Oxford NOTECHS II measures the human contribution. It is generally agreed that a full assessment of theatre team performance requires evaluation of both technical and non-technical performance[14], [21], [32], [33]. Our findings suggest that Oxford NOTECHS II and glitch count are orthogonal in terms of what they measure, and therefore complement each other well in achieving this.

An encouraging finding is that the majority of operations were performed by well-coordinated and functioning teams. With regards to differences between the theatre sub-teams, we found that the surgical team varied the most and utilised more of the scale than the anaesthetic or nursing team. We consider that this is due to the cultural and functional differences between the teams. The surgical team tends to comprise team members who are classical external processors, and their behaviour is necessarily linked to clearly observable technical tasks. The nursing team behaviour scores varied less, which may be due to within-team diversity. The nature of their work means that one team member (the scrub nurse) is entirely dependent upon the wider nursing team to support them, and where the behaviour of individuals within the team is very different, the team scores can become equivocal. The anaesthetic team's scores varied the least of all. Their work often requires little communication with the wider team although anaesthetists and anaesthetic support staff are in frequent communication. They also tend to make and action decisions from internally derived situational considerations. This makes the rating of anaesthetic behaviour difficult and this was borne out in our Oxford NOTECHS II sub-category analysis. This finding reproduced those of our original NOTECHS study[14]. As before, we have not weighted the significance of one team's contribution to the overall score as it remains unknown what significance each team's contribution has on the overall safety of the operating theatre. Further investigation may reveal that some teams contribute more to overall theatre team safety than others.

Due to the study design we were able to compare mean Oxford NOTECHS II scores across different sites and surgical specialities. There was no difference between the four NHS sites where elective orthopaedics or the two sites where vascular/general surgery was performed. We did however find differences between surgical specialities, with elective orthopaedic teams demonstrating higher Oxford NOTECHS II scores. This finding may demonstrate differences in the team dynamics due to the nature of the operation – with some requiring more overt team coordination than others. The number of teams observed is relatively small, however, and further work is required to exclude the possibility that this finding is simply due to personality and leadership differences within the particular surgical teams observed.

This study has a number of limitations. It would have been desirable to rate series of operations using both the original Oxford NOTECHS and the new scale in parallel, to evaluate how well we had achieved improved scalability whilst maintaining concurrent validity. This proved impossible as our observers considered they would be unable to switch between the scales and remain objective, whilst providing additional NOTECHS-trained observers was impracticable. As noted above, our findings about average Oxford NOTECHS II scores for different types of surgery require confirmation, as does our conclusion that a single observer may be able to score NOTECHS II reliably, whilst the problem of scoring anaesthetic behaviour adequately within a whole-team scale requires further work. Neither Oxford NOTECHS II nor other attempts to score surgical team behaviour has yet demonstrated a clear ability to predict clinical outcomes: our findings suggest that a combination of Oxford NOTECHS II and glitch count is more likely to be able to achieve this than either scale alone, but numerous other factors, particularly around post-operative care, are likely to confound this analysis. Our aim here is the exploration and development of a usable scale, which is an important and necessary step towards this goal.

## Conclusion

In conclusion we have found that the new Oxford NOTECHS II has retained good levels of inter-rater reliability making it potentially reliable with a solo observer. Validation studies showed the expected association with WHO time-out performance, and an orthogonal relationship with glitch rate which is theoretically explicable and may be valuable in permitting the use of complementary tools for overall assessment of teams. It offers a higher degree of precision than the previous version of the approach, potentially providing greater sensitivity in measurement. We have also found it to provide a relevant insight into the variation of theatre team's non-technical skills performance across surgical disciplines and hospitals. The approach appears to be a useful tool for exploring surgical performance for future research studies, but also may have applications as a quality improvement tool in its own right.
