# Correction: Oxford NOTECHS II: A Modified Theatre Team Non-Technical Skills Scoring System

**DOI:** 10.1371/journal.pone.0100111

**Published:** 2014-06-10

**Authors:** The PLOS ONE Staff

There is an error in the co-author, Dr. Damien Griffin’s, first name. The correct name for this co-author is Dr. Damian Griffin.
